# Bringing Insight to Light: Developing Psychoeducation for Psychosis in an Adolescent Inpatient Unit

**DOI:** 10.1192/bjo.2026.11437

**Published:** 2026-06-30

**Authors:** Francess Doherty, Karolina Szczygiel, Parvathy Mohandas

**Affiliations:** 1 Belfast Health and Social Care Trust, Belfast, United Kingdom; 2 Northern Ireland Medical and Dental Training Agency, Belfast, United Kingdom

## Abstract

**Aims:**

Psychoeducation is a key evidence-based component of treatment for psychosis, improving insight, adherence, and relapse prevention. The Orygen Psychosis Psychoeducation framework “A Shared Understanding: Psychoeducation in Early Psychosis” emphasises accessible, developmentally appropriate resources for young people and carers. Beechcroft, the regional Tier 4 adolescent inpatient unit for Northern Ireland, regularly admits young people experiencing psychosis. In 2022, a Serious Adverse Incident (SAI) at Beechcroft identified that a young person and their family felt insufficiently informed about a diagnosis of first-episode psychosis. This highlighted a gap in psychoeducational provision within the service and prompted the development of structured, age-appropriate psychoeducation workbooks.

**Methods:**

In response to the SAI, two psychoeducation workbooks were developed collaboratively by the Beechcroft team: one for young people, and one for parents and carers. The young person’s workbook is designed for use during the early recovery phase of inpatient admission and includes psychoeducation on the nature of psychosis, risk factors, symptom recognition, relapse indicators, treatment options, and relapse prevention planning. Delivery typically occurs over 4–6 sessions by a doctor, or alternatively could be delivered by another member of the multidisciplinary team (MDT) with appropriate training. The carer version provides parallel content adapted for families, with additional emphasis on understanding psychosis, managing relapse, and addressing feelings of guilt or self-blame. The psychoeducation workbooks have been piloted in Beechcroft with 3 young people and their carers. Qualitative feedback was collated in order to evaluate the effectiveness of our project to date.

**Results:**

Feedback from young people indicated that the workbook improved their understanding of psychosis, enhanced motivation for recovery, and encouraged engagement with treatment planning. Carers described the psychoeducation sessions as empowering, reporting greater confidence in recognising early warning signs and knowing when to seek professional help. Parents expressed relief in understanding that they were not responsible for their child’s illness, which facilitated a more supportive family environment.

**Conclusion:**

The introduction of structured psychoeducation workbooks in Beechcroft has strengthened communication between professionals, young people, and families while embedding NICE and Orygen aligned best practice into routine care. Early feedback suggests that the intervention enhances illness understanding, relapse management, and familyconfidence. Future plans include formal evaluation using outcome measures and exploring adaptations for community use and general adult psychiatric settings. The project demonstrates how learning from an SAI can drive meaningful service improvement and promote recovery-oriented practice in adolescent mental health inpatient units.

